# The complete chloroplast genome sequence of *Meconopsis punicea*

**DOI:** 10.1080/23802359.2020.1829135

**Published:** 2020-10-21

**Authors:** Chenxiaoning Meng, Mei Yang, Zhongyuan Guo, Yaohua Liang

**Affiliations:** a Institute of Chinese Materia Medica, China Academy of Chinese Medical Sciences, Beijing, China; b Bioduro, Beijing, China

**Keywords:** *Meconopsis punicea*, chloroplast genome, phylogenetic analysis

## Abstract

Meconopsis Vig. is a genus possessing important medicinal and ornamental values in the Papaveraceae. Many species in this genus are commonly used in traditional Tibetan medicines over thousands of years. In this study, we reported the complete chloroplast genome sequence of *Meconopsis punicea*. Total lengths of the chloroplast genomes were 152,933 bp. The genome had typical quadripartite structure, LSC region (83,031 bp) and SSC region (17,920 bp) were separated by a pair of IRs (25,991 bp), respectively. Moreover, they were composed of 131 genes, including 86 protein coding genes, 37 tRNA genes, 8 rRNA genes and one pseudogene. Phylogenetic analysis based on complete chloroplast genomes showed that *M. integrifolia* had closer relationship with *M. punicea*; meanwhile, *Meconopsis* was closely related to *Papaver* in Papaveraceae.

The genus Meconopsis Vig. belongs to the family Papaveraceae, comprises about 49 species in the world. Most of the species in this genus are distributed in eastern Asia and Western Europe. Many species are well-popular by local people due to their economic and ornamental values with blue flowers, such as *M. grandis*, *M. racemosa*, and *M. horridula*; thus, they are figuratively called ‘Himalayan blue poppies’. However, some others, such as *M. lancifolia*, *M. quintuplinervia*, *M. punicea*, and *M. integrifolia*, have purple, red, or yellow flowers. Some species of them have been widely used in traditional Tibetan medicine to treat inflammation, pain etc. However, for such an important genus, most of the studies focused on their chemical compositions; moreover, although there were some studies on molecular biology, there was nearly no report of complete chloroplast genome of Meconopsis species, except *M. racemose*. Here, we reported the complete chloroplast genome sequences of *Meconopsis punicea*, so as to reveal phylogenetic relationships between the species and related group in Papaveraceae.

Fresh and clean leave materials of *M. punicea* was collected separately from Hongyuan county (N32°43′05.99″, E102°34′03.57″) in Sichuan province, China. *Meconopsis punicea* represents an excellent model for understanding how different evolutionary forces have sculpted the variation patterns in the genome during the process of population differentiation and ecological speciation (Neale and Antoine 2011). Meanwhile, the voucher specimens with flowers (ZMXX001) were collected and deposited at the Herbarium of Institute of Chinese Materia Medica, China Academy of Chinese Medical Sciences. Total genomic DNA were extracted from fresh leaves by using the improved CTAB method (Doyle 1987). The obtained DNA was fragmented to construct a paired-end library with an insert-size of 300 bp and the genome sequencing were performed using HiSeq2000 (Novogene, Tianjin, China) platform. The raw data was filtered using Trimmomatic v.0.32 with default settings. We used the software MITObim 1.8 (Hahn et al. 2013) and metaSPAdes (Nurk et al. 2017) to assemble chloroplast genomes with the cp genome of closely related species *Meconopsis racemosa* (NC_039625) as the reference. Finally, we annotated the chloroplast genome using the software DOGMA (Wyman et al. 2004) with the cp genome of *Meconopsis racemosa* (NC_039625) as the reference genome and then corrected the results using Geneious 8.0.2 (Campos et al. 2016) and Sequin 15.50 (http://www.ncbi.nlm.nih.gov/Sequin/).

The annotated chloroplast genomes of *M. punicea* was submitted to the GenBank under the accession number of MK533648. Total lengths of the chloroplast genomes were 152,933 bp. The genome had typical quadripartite structure, LSC region (83,031 bp) and SSC region (17,920 bp) were separated by a pair of IRs (25,991 bp), respectively. Moreover, they were composed of 131 genes, including 86 protein coding genes, 37 tRNA genes, 8 rRNA genes, and 1 pseudogene.

To further investigate its taxonomic status, a maximum-likelihood (ML) tree was constructed based on complete chloroplast genome sequences using MEGA 7.0 (Kumar et al. 2016) with 1000 bootstrap replicates. We used the complete chloroplast genomes sequence of *M. punicea* and 13 other related species to construct phylogenetic tree. The 14 chloroplast genome sequences were aligned with MAFFT (Katoh and Standley 2013), and then the Maximum-likelihood (ML) tree was constructed. The phylogenetic analysis revealed that *M. punicea* and *M. integrifolia* clustered together as sisters to other related species (Figure 1).

**Figure 1.**
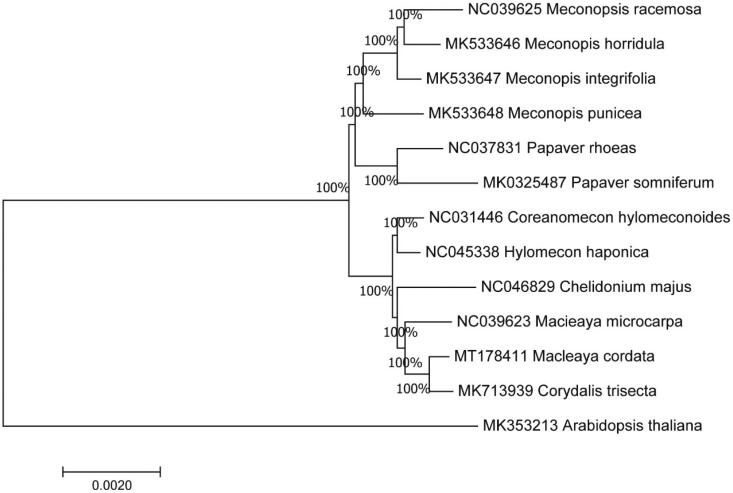
Maximum likelihood phylogenetic tree of *Meconopsis punicea* and other related species based on the complete chloroplast genome sequence.

## Data Availability

The data that support the findings of this study are openly available in GenBank at https://www.ncbi.nlm.nih.gov, reference number MK533648.
